# Plasma-Ionized Magnesium in Hospitalized Horses with Gastrointestinal Disorders and Systemic Inflammatory Response Syndrome

**DOI:** 10.3390/ani12121479

**Published:** 2022-06-07

**Authors:** Julia Sanmartí, Lara Armengou, Lucas Troya-Portillo, José Ángel Robles-Guirado, Anna Bassols, José Ríos, Eduard Jose-Cunilleras

**Affiliations:** 1 Servei de Medicina Interna Equina, Fundació Hospital Clínic Veterinari, Universitat Autònoma de Barcelona, Bellaterra, 08193 Barcelona, Spain; julia.sanmarti@uab.cat (J.S.); lara.armengou@uab.cat (L.A.); lucas.troya@uab.cat (L.T.-P.); 2 Departament de Medicina i Cirurgia Animals, Facultat de Veterinària, Universitat Autònoma de Barcelona, Bellaterra, 08193 Barcelona, Spain; 3 Departament de Bioquímica i Biologia Molecular, Facultat de Veterinaria, Universitat Autònoma de Barcelona, Bellaterra, 08193 Barcelona, Spain; josea_roblesg@hotmail.com (J.Á.R.-G.); anna.bassols@uab.cat (A.B.); 4 Department of Clinical Farmacology, Hospital Clinic, Medical Statistics Core Facility, Institut d’Investigacions Biomèdiques August Pi i Sunyer (IDIBAPS), 08036 Barcelona, Spain; jose.rios@uab.cat; 5 Biostatistics Unit, Faculty of Medicine, Universitat Autònoma de Barcelona, 08193 Barcelona, Spain

**Keywords:** hypomagnesemia, hypocalcemia, gastrointestinal disorders, electrolytes, equine

## Abstract

**Simple Summary:**

Gastrointestinal diseases are the most prevalent diseases in hospitalized horses. One of the predisposing factors of decreased intestinal motility in horses with colic could be low plasma calcium concentration and related magnesium imbalances. Magnesium and calcium disturbances can predispose to decreased intestinal motility in horses with colic after abdominal surgery. Plasma ionized magnesium concentrations were measured in hospitalized horses with different types of gastrointestinal disorders (i.e., colic), horses with non-gastrointestinal diseases, horses with systemic inflammatory conditions and control horses; our results allowed us to conclude that hypomagnesemia was more prevalent in horses with obstructive gastrointestinal lesions. Surprisingly, no association was found with systemic inflammatory conditions nor with mortality. These valuable results suggest to us that magnesium concentrations in horses presenting obstructive colic should be monitored. Further research is needed to evaluate the effects of intravenous magnesium supplementation in horses with gastrointestinal diseases.

**Abstract:**

Magnesium disorders in horses with gastrointestinal disorders or systemic inflammatory response syndrome (SIRS) are scarcely characterized. The purpose of the study was to explore the association of magnesium disorders with diagnosis, SIRS and mortality in horses admitted to a referral equine hospital. In total, 75 sick horses were included in an observational prospective study and classified as: obstructive (*n* = 17), inflammatory (*n* = 10) and ischemic gastrointestinal disorders (*n* = 12), and other non-gastrointestinal systemic disorders (*n* = 36). All sick horses were also divided according to the presence (*n* = 26) or absence of SIRS, and survival to discharge from hospital (survivors (*n* = 61) and non-survivors (*n* = 14). In addition, 26 horses were included as controls. On admission, mean (iMg) (95% confidence interval (CI)) in the SIRS group (0.47 (0.43–0.50 mmol/L)) was within the normal range (0.4–0.6 mmol/L). The obstructive group had lower (iMg) compared to the control group (0.44 (0.38–0.51 mmol/L) vs. 0.56 (0.50–0.61 mmol/L); *p* = 0.001). In total, 8 out of 17 (47%) horses with obstructive lesions presented with hypomagnesemia compared to controls (4% (1/26)) (*p* = 0.001). In conclusion, hypomagnesemia was more prevalent on admission in horses in the obstructive group, and to a lesser extent, in the inflammatory and ischemic groups. In contrast to human ICU patients, the proportion of hospitalized horses with hypomagnesemia was not associated with mortality.

## 1. Introduction

In horses and other mammals, plasma magnesium (Mg) is found in three forms: the physiologically active ionized (iMg) form (60%), bound to proteins (albumin, globulins) (30%) and bound to weak acids (bicarbonate, citrate, sulfate, phosphate, etc.) (10%) [1,2,3,4,5,6,7]. Analysis of the ultra-filtrates (complexed Mg + iMg) does not distinguish the truly ionized form from the one bound to organic and inorganic anions [8]. Because the concentrations of these ligands can vary significantly in numerous pathological states, it is desirable to directly measure the concentrations of iMg [8,9]. The reported physiologic total serum magnesium (tMg) concentrations in horses range from 1.4 to 2.2 mg/dL (0.6 to 0.9 mmol/L), and for ionized magnesium (iMg), they range from 0.9 to 1.5 mg/dL (0.42 to 0.6 mmol/L) [1,2,3,4,5,6,7,10]. Clinical signs of hypomagnesemia are rarely reported in horses but include weakness, muscle fasciculations, ventricular arrhythmias, seizures, ataxia and coma [11].

In human medicine, the common causes of hypomagnesemia due to gastrointestinal loss are diarrhea (acute or chronic), vomiting and malabsorption (Crohn disease, celiac disease, small bowel resection or bypass) [12]. In healthy people, total plasma magnesium concentrations range from 0.7 to 1.1 mmol/L [13]. Several studies found associations between Mg concentrations and various gastrointestinal (GI) disorders in horses. Hypomagnesemia (tMg) has been reported in 48.7% of hospitalized horses [14], in 17% (tMg) and 54% (iMg) of equine surgical colic patients [15] and in 78% (iMg) of horses with enterocolitis [10]. Horses with multiple organ failure or dysfunction were 2.3 times more likely to present with hypomagnesemia and have longer hospital stays [14]. Decreased (iMg) is a cause of persistent hypocalcemia through impaired PTH secretion, end-organ parathyroid hormone (PTH) responsiveness and decreased synthesis of 1,25-dihydroxyvitamin D3 that has been observed in horses and humans [16].

Systemic inflammatory response syndrome (SIRS) is the clinical manifestation of dysregulated immune responses to infectious or noninfectious stimuli [17]. In human clinical trials, SIRS was defined by the presence of at least two of the following clinical signs: (1) hypothermia or hyperthermia, (2) tachycardia, (3) tachypnea or hypocapnia and (4) leukocytosis, leukopenia or an increased number of immature leukocyte forms [17]. This definition from human medicine was adapted by Roy et al. (2017) to develop a scoring system in horses with SIRS [18]. SIRS is associated with an increased risk of death in adult horses presenting with acute gastrointestinal disorders [19], and subclinical hypomagnesemia could increase the severity of SIRS [11]. Based on previous human and veterinary studies, our hypotheses were: (1) hypomagnesemia is more frequent in horses with GI disorders and SIRS; and (2) ionized hypomagnesemia is associated with outcome.

The purpose of this study was to measure iMg concentration in adult horses on hospital admission and over time (day 0, day 2–4) and detect hypomagnesemia, hypocalcemia and concomitant hypomagnesemia with hypocalcemia, and to identify the associations of plasma iMg concentration with GI disorders, SIRS (with or without GI disorders) and survival.

## 2. Materials and Methods

### 2.1. Animals

Adult horses referred to the Unitat Equina, Fundació Hospital Clínic Veterinari, Universitat Autònoma de Barcelona, Spain, from May 2019 to July 2020 for GI or non-GI systemic/inflammatory disorders were included in the study. In addition, adult horses admitted for elective surgical procedures and a group of 14 healthy adult mares from the Equine Reproduction Unit of the Universitat Autònoma de Barcelona were also included as a control group. For the purpose of this study, adult horses were considered >1 year of age.

Horses with GI disorders were classified into 3 groups: obstructive, inflammatory and ischemic. All the other admitted horses were classified as non-GI systemic disorders. Donkeys, mules and neonatal foals were excluded from the study. Horses included in this study did not receive any magnesium supplementation, either as oral magnesium sulphate laxatives or supplemented as injectable magnesium sulphate to the intravenous (IV) fluid therapy. Horses received calcium supplementation in IV fluid therapy if ionized hypocalcemia (iCa < 1.4 mmol/L) was present. Horses that required laxatives had sodium sulphate administered by nasogastric tube. Outcome was defined as either survival to discharge from the hospital or non-survivors, and horses that were euthanized due to economic constraints were not included in this study.

Horses were classified in the SIRS group if they presented 2 or more of the following 4 criteria on admission: (1) hyper- or hypothermia (rectal temperature > 38.5 °C or <37 °C), (2) tachycardia (heart rate > 52 beats/min), (3) tachypnea (respiratory rate > 20 breaths/min) or (4) leukopenia or leukocytosis (white blood cell counts < 5000 cell/µL or >12,500 cell/µL) [18].

For the purpose of this study, survivors were defined as those discharged from the hospital, and non-survivors were those horses that required euthanasia due to poor prognosis and significant deterioration despite aggressive medical treatment and occasionally a horse with rapid deterioration and death. If any horse was euthanized for financial constraints, it was excluded from the analysis of survival.

### 2.2. Blood Sampling

The initial blood sample was collected on admission before any treatment was administered in the hospital, and afterward, daily samples were also collected if the horse was hospitalized. Whole blood samples were collected in lithium heparin tubes. If the sample could not be processed immediately, it was kept refrigerated at 4 °C until processed. A maximum of 24 h delay between sampling and analysis was considered acceptable to be included in the database. This was decided a priori based on previously published research that confirmed (iMg) stability up to 48 h stored at 4 °C in whole blood lithium heparin tubes [19]. The same processing method was applied to all samples in order to standardize the sampling procedure. All measurements were performed with a benchtop co-oximetry and electrolytes analyzer by direct ion-selective electrode method (Stat Profile^®^ Prime Plus Vet Analyzer, Nova Biomedical, Waltham, MA, USA). According to the manufacturer specifications, the calibration, tolerance ranges and coefficients of variation were: (i) two-point calibrations (set points: iCa: 1.04 and 1.92 mmol/L; iMg: 0.5 and 1.5 mmol/L) with predefined limits to ensure that sensors operate within a defined calibration range; (ii) iMg sensor range 0.1–1.5 mmol/L, intra-assay coefficient of variation (%/SD): 2.0%/0.03 mmol/L and day-to-day imprecision (%/SD): 4.0%/0.05 mmol/L; and (iii) iCa sensor range 0.1–2.7 mmol/L, intra-assay coefficient of variation (%/SD): 2.0%/0.05 mmol/L; and day-to-day imprecision (%/SD): 4.0%/0.07 mmol/L.

### 2.3. Data Analysis

Data were summarized by mean and 95% confidence interval (95% CI) for quantitative variables and absolute frequency and percentage for qualitative variables, except for age at admission, described by median and 95%CI. Plasma concentrations of ionized magnesium (iMg) and calcium (iCa) were measured over time (admission and 2–4 days) to establish an association with SIRS, diagnosis category (obstructive, inflammatory, ischemic, non-GI disorder or control) or outcome. In each case, differences were analyzed within a group over time (intra-group) and between groups at a single time point (inter-group differences). Ionized hypomagnesemia was defined as plasma (iMg) below 0.4 mmol/L and ionized hypocalcemia as plasma (iCa) below 1.4 mmol/L [11,20].

The association of SIRS, diagnosis and outcome with (iMg) and (iCa), as dependent variables, was explored by generalized estimation equation (GEE) models using an autoregressive (1) to account for intra-subject variability. GEE models extend the generalized linear models to allow for analysis of repeated measurements in longitudinal data sets [21]. All GEE models included group (SIRS, diagnosis or outcome), supplementation of calcium in IV fluids, time (admission and follow-up at 2–4 days) and interaction of time by group. Age was not included in the GEE models (as a possible confounder), given that it was not significantly different between groups. Pair-wise comparisons with the control group were adjusted for type I error by Bonferroni’s criteria. Comparison between groups for continuous variables was performed with the Mann–Whitney U-test (for 2 groups) or the Kruskal–Wallis test (for ≥3 groups). Fishers’ exact test was used to test for differences in the proportions of hypomagnesemia, hypocalcemia or both and diagnosis, outcome or SIRS groups.

All statistical analyses were performed using a statistical software package (SPSS version 26, IBM Corp, Armonk, NY, USA), and in all statistical analyses, a two-sided type I error was set at 5%.

## 3. Results

### 3.1. Patient Characteristics and Diagnostic Classification

One hundred and one adult horses of various breeds were included in the study. The distribution of gender was 44 females (43.6%), 32 geldings (31.7%) and 25 stallions (24.7%). In total, 31 (30.7%) horses were Andalusians, 29 (28.7%) crossbred, 15 (14.9%) Arabians, 12 (11.9%) mixed European saddle breeds, 4 (0.04%) Friesians, 4 (0.04%) draft breeds, 3 (0.03%) thoroughbred and 3 (0.03%) crossbred ponies. Age distribution within groups is summarized in Table 1. The median age in SIRS (10.9 years old) and non-SIRS (11.9 years old) was comparable (*p* = 0.715).

Horses were classified into five diagnostic groups: obstructive (*n* = 17), inflammatory (*n* = 10), ischemic (*n* = 12), non-GI systemic disorders (*n* = 36) and control (*n* = 26). The obstructive group included horses with stomach, caecum and large colon impactions and large colon displacements successfully treated medically. The inflammatory group included duodenitis/proximal jejunitis and enterocolitis of different etiologies and severities. All horses in the ischemic group were treated surgically due to lesions, such as small intestinal inguinal hernia, small intestinal and large colon volvulus, small intestine foramen epiploic entrapment, jejunal-ileocecal intussusception and small intestine mesenteric hernia. The comparison group with non-GI systemic disorders included: respiratory (i.e., pneumonia and pleuritis), hepatic (i.e., cholangiohepatitis), reproductive (i.e., septic metritis and dystocia), musculoskeletal (i.e., rhabdomyolysis and severe lacerations/wounds) and ophthalmologic diseases (i.e., severe corneal ulceration or recurrent uveitis). The control group included healthy horses admitted for elective orthopedic surgery (i.e., osteochondritis dissecans), castrations, diagnostic imaging workup (i.e., echocardiography, scintigraphy), healthy mares accompanying foals and healthy horses from the Equine Reproduction Unit.

### 3.2. Plasma-Ionized Magnesium Concentrations

**Figure 1:**
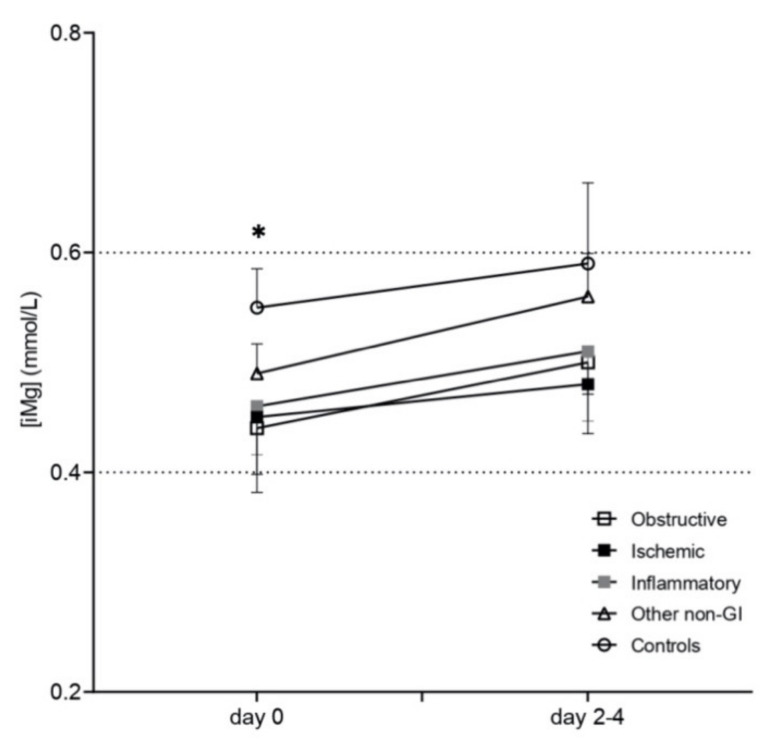
Mean (±2SE) (iMg) (mmol/L) over time (plotted in two time points: day 0 and day 2–4) in sick adult horses (open square = obstructive, black square = ischemic, gray square = inflammatory, open triangle = non-GI disorders, open circles = controls). Horizontal dotted lines from the left Y axes show the reference range for (iMg) in adult horses. * obstructive and ischemic are significantly different from control group.

Mean (95% CI) plasma (iMg) in the control group on admission was 0.55 (0.51–0.58 mmol/L). Significant differences were detected in plasma (iMg) relative to control horses: obstructive group (0.44 (0.38–0.51); *p* = 0.001) and ischemic group (0.45 (0.39–0.50 mmol/L); *p* = 0.002). Plasma (iMg) did not significantly change over time (day 0 vs. days 2–4) within each diagnostic group (Figure 1). Hypomagnesemia was more frequent in horses with obstructive lesions (8/17 horses (47%); *p* = 0.001) and, to a lesser extent, in the ischemic group (4/12 horses (33%); *p* = 0.02) compared to controls (1/26 (4%); Table 1)).

Based on the equine SIRS criteria [18], there were 26 horses in the SIRS group and 49 in the non-SIRS group. In addition, there were 61 survivors and 14 non-survivor horses (12 humane euthanasia and 2 deaths). The proportion of horses with SIRS was not significantly different between the diagnostic groups: obstructive 7/17 (41%), inflammatory 4/10 (40%), ischemic 5/12 (33%) and non-GI disorders 10/36 (28%). No differences were observed on admission in SIRS vs. non-SIRS horses in the prevalence of hypomagnesemia (<0.4 mmol/L), hypermagnesemia (>0.6 mmol/L) and altered magnesium (<0.4 or >0.6 mmol/L) (Table 1).

**Figure 2:**
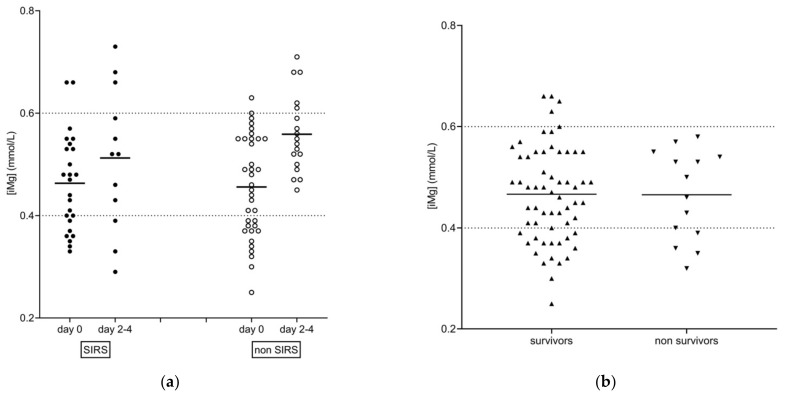
Scatter dot plot of (iMg) (mmol/L) in sick adult horses: (**a**) distributed in columns based on SIRS (solid circles) and non-SIRS (open circles) over time (day 0, day 2–4) and (**b**) distributed in columns based on survivors (solid upward triangles) and non-survivors (solid downward triangles). Horizontal solid lines indicate mean (iMg). Horizontal dotted lines from the left Y axis show the reference range for (iMg) in adult horses.

Mean (95% CI) (iMg) on admission in survivors (0.49 (0.47–0.51 mmol/L)) compared to non-survivors (0.47 (0.41–0.52 mmol/L)) was significantly different (*p* = 0.036). Moreover, significant increases in (iMg) were seen over time in both survivors (0.52 (0.50–0.53 mmol/L); *p* = 0.04) and non-survivors (0.58 (0.49–0.67 mmol/L); *p* = 0.011). However, the proportion of hypomagnesemia ((iMg) < 0.4 mmol/L) in survivors (17/87; 20%) and non-survivors (4/14; 29%) was not different (*p* = 0.48). The distribution of all data upon admission in SIRS, non-SIRS horses and according to the outcome, is presented in Figure 2.

### 3.3. Plasma-Ionized Calcium Concentrations

Mean (95% CI) (iCa) on admission was significantly lower in horses with GI disorders compared to healthy horses (1.53 (1.50–1.57 mmol/L)). Horses in the obstructive, inflammatory and ischemic groups had mean (iCa) (95%CI) 1.45 (1.39–1.50), (*p* = 0.004); 1.41 (1.29–1.54), (*p* = 0.012) and 1.41 (1.34–1.47 mmol/L) (*p* = 0.001), respectively (Figure S1). Horses in the non-SIRS group significantly increased plasma (iCa) over time: 1.48 (1.46–1.51) on admission and 1.57 (1.56–1.59 mmol/L) by 2–4 days of hospitalization (mean (95% CI); *p* < 0.001). In contrast, plasma (iCa) in horses with SIRS remained low and did not significantly change over time from day 0 (1.46 (1.40–1.52 mmol/L)) to days 2–4 (1.45 (1.36–1.55 mmol/L); *p* = 0.8)) (Figure S2). Combined hypomagnesemia and hypocalcemia were significantly more prevalent in the obstructive group, where 5/17 (29.4%) of the horses had both abnormalities compared to the control group (1/26; *p* = 0.026). In addition, we did not observe an association of (iCa) or (iMg) with blood pH (Figures S3 and S4), and there was no statistically significant difference in pH between the groups: control 7.43 (7.41; 7.45), inflammatory 7.40 (7.33; 7.47), obstructive 7.44 (7.40; 7.48), ischemic 7.44 (7.39; 7.49) and non-GI disorders 7.42 (7.41; 7.44) (mean; 95%CI of mean).

## 4. Discussion

The main results of this study were: (1) horses with obstructive and ischemic gastrointestinal disease presented with lower (iMg) and lower (iCa) compared to healthy horses; (2) the obstructive group had more horses with hypomagnesemia as well as simultaneous hypomagnesemia and hypocalcemia; (3) no association of plasma (iMg) or (iCa) with SIRS was detected; and (4) this population of horses did not have significant association of (iMg) with outcome.

In this study, horses with gastrointestinal disease had low (iMg) and low (iCa) more often compared to healthy horses. In previous studies, hypomagnesemia and hypocalcemia were present more often in horses with enterocolitis [10]. Similarly, horses with strangulating lesions of the GI tract had significantly lower preoperative (iMg), and those with postoperative ileus had lower (iMg) during hospitalization [15]. Starvation or anorexia and poor intestinal absorption due to an inflamed bowel could contribute to hypomagnesemia in horses with inflammatory intestinal problems or ileus [11]. In addition, intestinal wall inflammation in horses with inflammatory and ischemic colic predisposes to bacterial translocation and absorption of endotoxins from the intestinal lumen [22]. Increased plasma endotoxin concentrations in horses with enterocolitis and increased pro-inflammatory mediators may indirectly and variably suppress PTH secretion in horses [10]. In this study, a combination of an inflamed intestinal wall, increased concentrations of inflammatory cytokines and starvation could all potentially contribute to hypomagnesemia in horses with GI disorders.

In the present study, horses in the obstructive group had hypomagnesemia or simultaneous hypomagnesemia and hypocalcemia more frequently. In dogs, the most common cause of concurrent hypomagnesemia and hypocalcemia is gastrointestinal disease [23]. In contrast to other minerals, intestinal Mg absorption is poorly regulated. Extracellular Mg is not under tight hormonal homeostatic control like calcium is, and plasma concentrations depend on gastrointestinal absorption, renal excretion and bone exchange [24,25,26]. In the present study, horses with progressive obstructive GI disorders often presented on admission with a history of partial or complete anorexia for a few days, which could partially explain the observed hypomagnesemia in this group. In our referral population of horses with gastrointestinal disorders, it would be unusual for our referring veterinary surgeons to administer fluids in a field setting simply for anorexia without significant dehydration. A more frequent finding of hypomagnesemia in hospitalized horses with obstructive and ischemic GI lesions should be considered when designing fluid therapy for these cases.

Ionized magnesium concentrations were not associated with the presence of SIRS on admission in this study. SIRS is the clinical manifestation of dysregulated immune responses to infectious or noninfectious stimuli [17], and it was suggested that SIRS should replace the widely used term endotoxemia to describe the clinical status of horses with severe colic [27]. This syndrome is clinically relevant in horses with colic, and a closer analysis using broad diagnostic categories revealed that SIRS was a predictor of poor outcome in acute gastrointestinal emergencies [18]. Although SIRS is directly proportional to disease severity, it is multifactorial and not exclusively related to GI illnesses.

In our study, plasma (iMg) and (iCa) were not clearly associated with outcome. Although (iMg) was statistically different, the magnitude of this difference was not considered clinically relevant. Hypomagnesemia is common in critically ill human patients, with a prevalence estimated to be between 8% and 30% [12,13]. There is strong, consistent clinical evidence, largely from observational studies, showing that hypomagnesemia is significantly associated with increased need for mechanical ventilation, prolonged ICU stay and increased mortality [9,28]. In studies of critically ill dogs and cats that determined plasma (tMg), hypomagnesemia and hypermagnesemia were associated with prolonged hospitalization (*p* < 0.05) and increased mortality (OR 2.6) [29,30]. Similarly, horses with hypomagnesemia on admission (plasma (tMg)) were more likely to be hospitalized longer (OR 1.58) [14]. However, divergent results have been published in the equine literature regarding the associations of plasma (tMg) and (iMg) with survival. In septic and sick non-septic foals, no associations were found between ionized hypomagnesemia and outcome [16]. In horses that had colic surgery, total and ionized (Mg) were not found to be associated with hospitalization time, complications or survival [15]. Paradoxically, Johansson et al. found that horses with hypomagnesemia (tMg) were more likely to survive [14]. Contrary to human studies, and in agreement with some equine hospital studies [15,16], in our group of horses, (iMg) was not associated with outcome. This could be partly explained by differences in the pathophysiology and severity of GI diseases in this study relative to those in ICU human patients. Decreased plasma (iMg) is mainly explained by three mechanisms in all species: decreased intake or poor absorption, redistribution within the organism and excess excretion. The diet in horses is rich in Ca and Mg, and horses depend on significant renal excretion of Ca (and likely Mg) to maintain homeostasis, whereas humans ingest a diet with lower Ca and Mg, have lesser GI absorption of these and depend much less on renal excretion to maintain homeostasis [31]. In addition, the differences in iMg pathophysiology between human and equine patients are also related to the disorders diagnosed most frequently. There is some evidence of magnesium dysregulation in human patients with certain neoplasias and in those with insulin disorders. Both of these are much less common in equine rather than human patients [32].

Horses with colic referred to an equine hospital normally present with dehydration, decreased peripheral perfusion and decreased glomerular filtration that could influence (iMg) [14]. Plasma (tMg) depends on protein concentration. Magnesium is bound to total protein (approximately 32%), about three-fourths is bound to albumin (25%) and the rest to globulins (8%) [33]. As 30% of plasma magnesium is bound to albumin, measuring total plasma magnesium may provide a spuriously low value in patients with hypoalbuminemia [11]. Plasma (iMg) also depends on acid-base status; acidosis increases (iMg) and alkalosis decreases it [34,35]. This is clinically relevant, as the conditions associated with alkalosis (i.e., hypochloremic alkalosis in horses with chloride loss due to duodenitis/proximal jejunitis) can lead to low (iMg) concentrations, with potential clinical signs of hypomagnesemia despite normal (tMg) [34]. In the present study, we did not observe an association between pH and (iMg), and the proportion of sick horses with increased pH and low (iMg) was relatively small. In horses with surgical colic, if (tMg) were examined rather than (iMg), 37% fewer horses with true hypomagnesemia would be detected [15]. Therefore, it is important to measure (iMg) rather than (tMg) in horses with hypoalbuminemia and acid-base disturbances.

This study had several limitations. Urine samples were not obtained, and measurement of urinary fractional excretion of magnesium (FMg) was not performed in these hospitalized horses. Renal diseases and acid-base disturbances that result in excessive urinary losses could also lead to hypomagnesemia because Mg is filtered through the glomerular membrane [25,36,37]. FMg would have helped us to assess the amount of magnesium lost through the kidney to better quantify the specific intestinal losses in horses with hypomagnesemia [1,10,38]. We did not measure FMg because the vast majority of horses in the study did not present with kidney injury, and intravenous fluid therapy could alter the results of fractional excretions by induced diuresis. Another limitation of the study was the concomitant calcium supplementation in intravenous (IV) fluid therapy in horses with confirmed hypocalcemia. Although statistical analysis did correct for the effect of calcium supplementation, horses in all gastrointestinal disease groups were treated with IV and enteral fluid therapy. In our hospital, we do not routinely treat horses with obstructive colic with oral magnesium sulfate laxatives because we have good treatment success using isotonic (0.6% NaCl and 0.3% KCl) enteral fluid therapy. It is possible that the fluid therapy being used could influence (iMg) or (iCa) over time (2–4-day samples). Samples on admission would have been less likely affected because few horses were treated with large volumes of fluid therapy (IV or enteral) before referral.

## 5. Conclusions

Hypomagnesemia was more prevalent in horses in the obstructive group (~50%) and, to a lesser extent, in the ischemic group (~30%) compared to healthy horses (4%), possibly related to fasting and the pathophysiology of the gastrointestinal disease. When designing IV fluid therapy for horses with obstructive and ischemic GI lesions, magnesium supplementation should be considered, given the higher proportion of cases with hypomagnesemia. In contrast to human ICU patients, outcome was not associated with hypomagnesemia. The role of ionized magnesium in horses with gastrointestinal disorders warrants further research.

## Figures and Tables

**Table 1:** Age and proportion of horses with hypomagnesemia (↓(iMg)), hypermagnesemia (↑(iMg)) and simultaneous hypomagnesemia and hypocalcemia (↓(iCa)) according to diagnostic and SIRS groups.

	Control (*n* = 26)	Obstructive (*n* = 17)	Inflammatory (*n* = 10)	Ischemic (*n* = 12)	Non-GI (*n* = 36)	SIRS (*n* = 26)	Non-SIRS (*n* = 75)
↓(iMg) *n* (%)	1 (3.9%)	8 (47.1%) *	3 (30%) *	4 (33.3%) *	5 (13.9%) *	7 (26.9%)	14 (18.7%)
↑(iMg) *n* (%)	9 (34.6%)	2 (11.8%)	0 *	0 *	2 (5.6%) *	2 (7.7%)	11 (14.7%)
↓(iCa) + ↓(iMg) *n* (%)	1 (3.9%)	5 (29.4%) *	2 (20%)	3 (25%)	2 (5.6%) ^$^	4 (15.4%)	9 (12%)
Age median (95%CI) (range)	7 (6–11) (2–26)	14 (11–19) (1.5–23)	9.5 (6–15) (4–32)	9 (4–11) (2–23)	12 (10–15) (2–26)	10 (10–12) (2–26)	11 (9–17) (1.5–21)

Note: number of horses in each group (*n*); percentage (%); * significantly different to control group; ^$^ significantly different to obstructive group. Adjusted *p*-value for multiple comparisons <0.0125 (Bonferroni’s method).

## Data Availability

The data presented in this study are available on request from the corresponding author.

## Supplementary Materials

The following supporting information can be downloaded at: https://www.mdpi.com/article/10.3390/ani12121479/s1, Figure S1: Mean (±2 SE) plasma (iCa) (mmol/L) over time (plotted in two time points: day 0 and day 2–4) in sick adult horses (open square = obstructive, black square = ischemic, gray square = inflammatory, open triangle = non-GI disorders, open circles = controls). Horizontal dotted lines from the left Y axes show the reference range for (iCa) in adult horses. * obstructive, ischemic and inflammatory are significantly different from control group; Figure S2: Scatter dot plot of plasma (iCa) (mmol/L) in sick adult horses: (a) distributed in columns based on SIRS (solid circles) and non-SIRS (open circles) over time (day 0, day 2–4); and (b) distributed in columns based on survivors (solid upward triangles) and non-survivors (solid downward triangles). Horizontal solid lines indicate mean (iCa). Horizontal dotted lines from the left Y axis show the reference range for (iCa) in adult horses; Figure S3: Relationship between pH and plasma (iMg) (mmol/L) in sick adult horses; Figure S4. Relationship between pH and plasma (iCa) (mmol/L) in sick adult horses.
