# Supplementary figures and images for: Plasma-Ionized Magnesium in Hospitalized Horses with Gastrointestinal Disorders and Systemic Inflammatory Response Syndrome
Source: Animals (Basel). 2022 Jun 7;12(12):1479. doi: 10.3390/ani12121479 (PMC9219524; doi:10.3390/ani12121479)

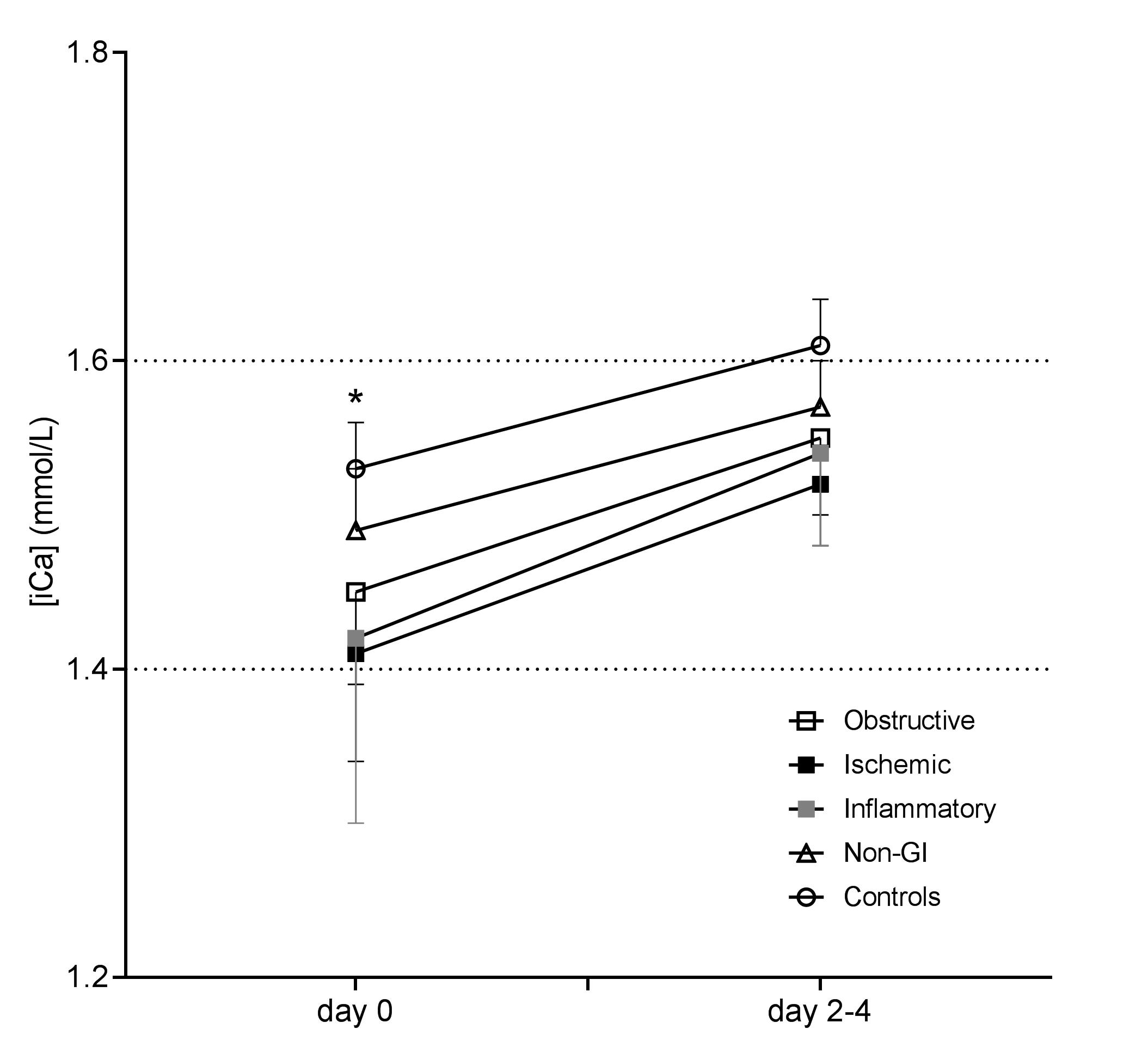

Supplement: Supplementary file 1 [file animals-12-01479-s001.zip › Figure S1.jpg]

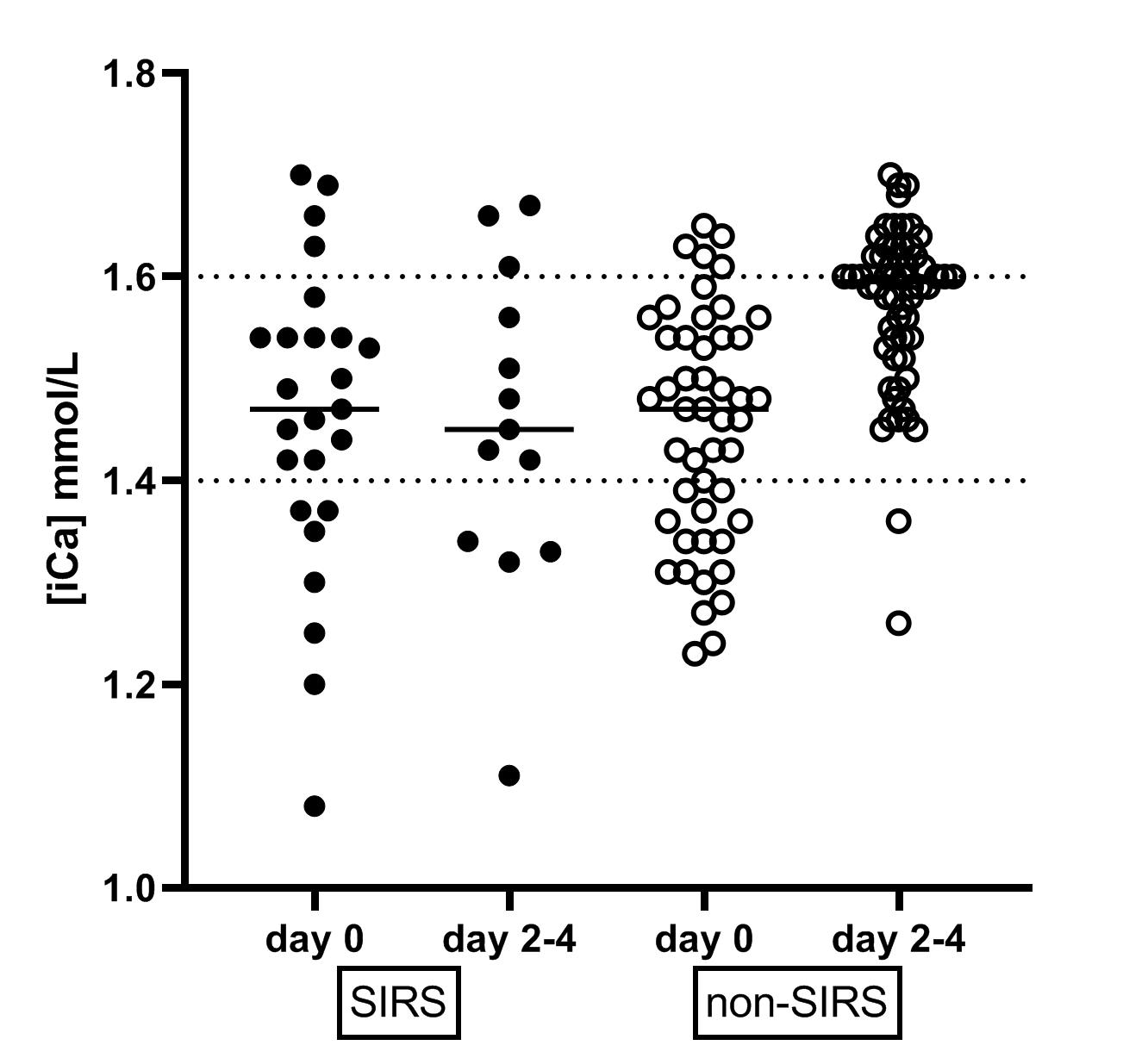

Supplement: Supplementary file 1 [file animals-12-01479-s001.zip › figure S2a.jpg]

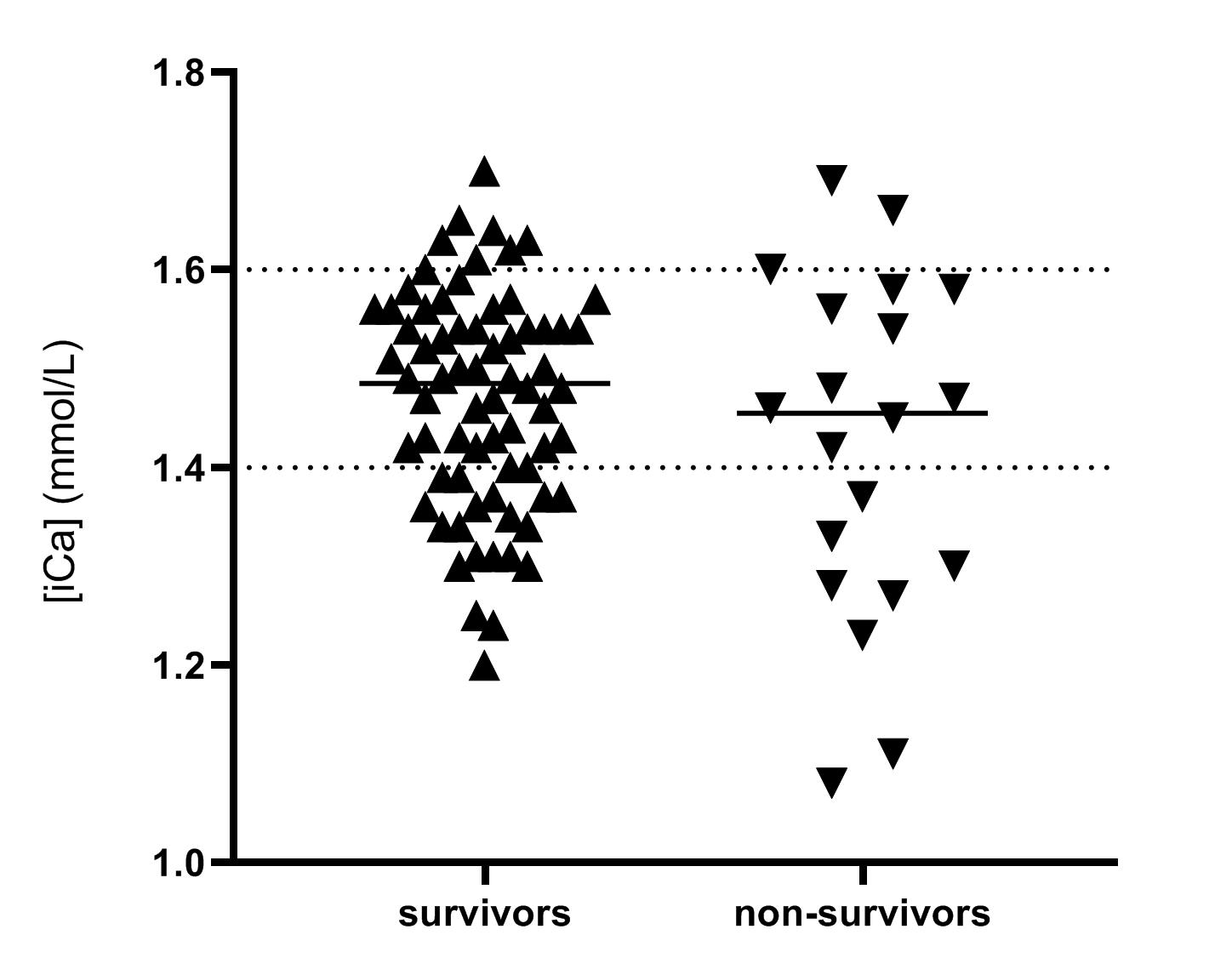

Supplement: Supplementary file 1 [file animals-12-01479-s001.zip › figure S2b.jpg]

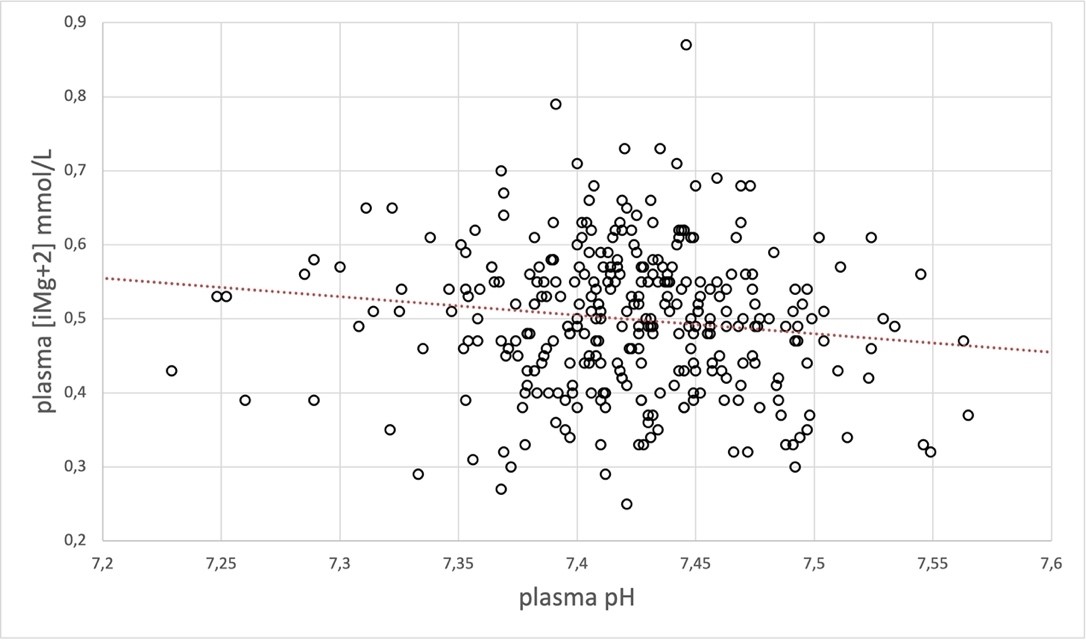

Supplement: Supplementary file 1 [file animals-12-01479-s001.zip › figure S3.jpg]

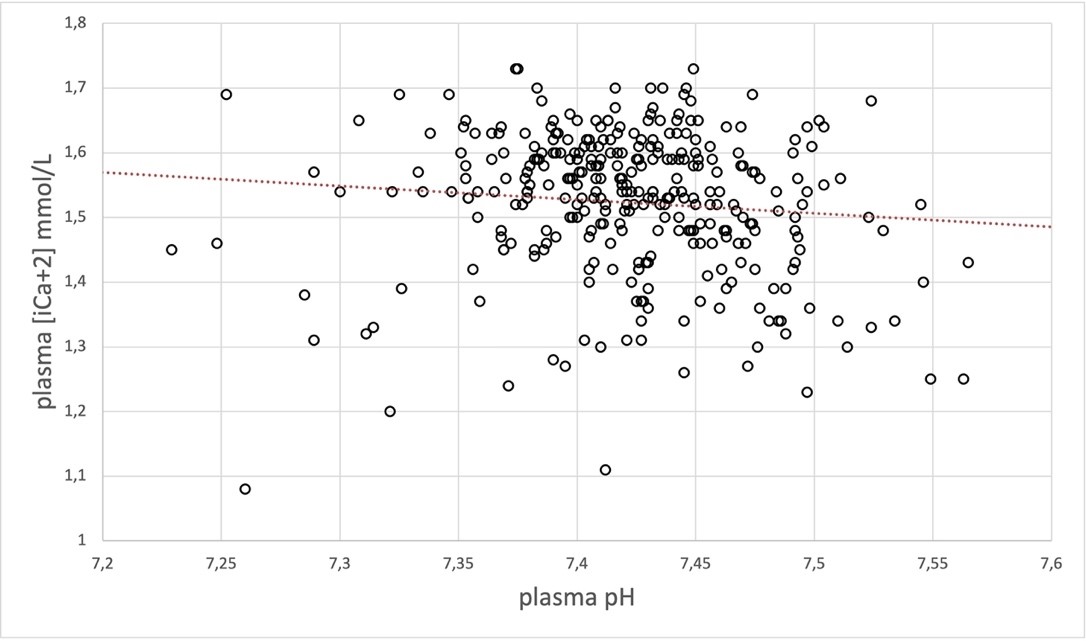

Supplement: Supplementary file 1 [file animals-12-01479-s001.zip › figure S4.jpg]
